# Quantitative detection of nitric oxide in exhaled human breath by extractive electrospray ionization mass spectrometry

**DOI:** 10.1038/srep08725

**Published:** 2015-03-04

**Authors:** Susu Pan, Yong Tian, Ming Li, Jiuyan Zhao, Lanlan Zhu, Wei Zhang, Haiwei Gu, Haidong Wang, Jianbo Shi, Xiang Fang, Penghui Li, Huanwen Chen

**Affiliations:** 1 Jiangxi Key Laboratory for Mass Spectrometry and Instrumentation, East China Institute of Technology, Nanchang, Jiangxi Province 330013, P. R. China; 2 State Key Laboratory of Environmental Chemistry and Ecotoxicology, Research Center for Eco-Environmental Sciences, Chinese Academy of Sciences, Beijing 100085, P. R. China; 3 National Institute of Metrology, Beijing 100013, P. R. China; 4 Department of Respiratory Medicine, The First Affiliated Hospital of Nanchang University, Nanchang, Jiangxi Province 330006, P. R. China

## Abstract

Exhaled nitric oxide (eNO) is a useful biomarker of various physiological conditions, including asthma and other pulmonary diseases. Herein a fast and sensitive analytical method has been developed for the quantitative detection of eNO based on extractive electrospray ionization mass spectrometry (EESI-MS). Exhaled NO molecules selectively reacted with 2-phenyl-4, 4, 5, 5-tetramethylimidazoline-1-oxyl-3-oxide (PTIO) reagent, and eNO concentration was derived based on the EESI-MS response of 1-oxyl-2-phenyl-4, 4, 5, 5-tetramethylimidazoline (PTI) product. The method allowed quantification of eNO below ppb level (~0.02 ppbv) with a relative standard deviation (RSD) of 11.6%. In addition, eNO levels of 20 volunteers were monitored by EESI-MS over the time period of 10 hrs. Long-term eNO response to smoking a cigarette was recorded, and the observed time-dependent profile was discussed. This work extends the application of EESI-MS to small molecules (<30 Da) with low proton affinity and collision-induced dissociation efficiency, which are usually poorly visible by conventional ion trap mass spectrometers. Long-term quantitative profiling of eNO by EESI-MS opens new possibilities for the research of human metabolism and clinical diagnosis.

Nitric oxide (NO) regulates a great number of biological processes, and its level in living organisms is very sensitive to physiological conditions^1^. Monitoring the concentration of exhaled nitric oxide (eNO) is of substantial interest in metabolism studies and clinical applications, because it is a non-invasive method to characterize airway pathology or pulmonary inflammation for various diseases, such as asthma, chronic obstructive pulmonary disease, lung diseases, etc.^2^,^3^,^4^,^5^,^6^,^7^,^8^,^9^,^10^ It also may be useful in identifying lung function deterioration in scleroderma^11^. The level of eNO from Hodgkin disease patients has been found higher than that after remission^12^,^13^.

To date, several analytical techniques including chemiluminescence^14^,^15^, electrochemical sensors^15^,^16^, diaminofluorescein (DAF-2 and DAF-2DA)^16^, and mass spectrometry^17^ have been used for eNO detection. For many years, the method based on measuring chemiluminescence in the photochemical reaction between NO and ozone was considered as the “gold standard” for eNO detection^18^. Reliable quantification of eNO by chemiluminescence is challenging and requires onsite calibration. Significantly different mean eNO values in human beings were reported by the research groups using different instrumentation for chemiluminescence detection^19^. Electrochemical sensing is more direct and simpler for operation, but the selectivity and the detection limit (~5 ppbv) of the currently available NO sensors require further development. Another limitation of electrochemical sensors is their low efficiency in NO multiple flow analysis (various expiratory flow rates for the sampling procedures)^20^. The combination of gas chromatography and mass spectrometry (GC-MS) is a powerful method for the sensitive and reliable detection of eNO in humans^17^. However, the throughput of GC-MS analysis is limited by the necessity of sample preparation, *e.g.*, breath condensation and water removal.

Recently, the possibility of direct human breath analysis was demonstrated using extractive electrospray ionization mass spectrometry (EESI-MS)^21^. In many cases, EESI-MS requires no/minimal sample pretreatment and allows sensitive detection of both volatile and non-volatile compounds in exhaled breath in real time with high throughput. By forming molecular complexes with silver cations (Ag^+^), analytes can be detected although they are difficult to be ionized with electrospray ionization (ESI) (e.g., CH_3_-S-CH_3_, CH_3_CN, etc.). This substantially enhances the molecular coverage of EESI-MS^22^,^23^. Improving the specificity of detection is of particular importance for small molecules such as NO, because the tandem MS analysis of NO ions (e.g., NO^+^) cannot be done with commonly used mass spectrometers. However, specific complex formation between NO molecules and Ag^+^ or other metal cations appears to be very difficult. This makes NO and similar compounds in breath and other matrices still invisible to EESI-MS.

In this work, an EESI-MS analytical method has been developed for the quantitative detection of eNO in humans based on the reaction between NO and 2-phenyl-4, 4, 5, 5-tetramethylimidazoline-1-oxyl-3-oxide (PTIO). Detection and quantification of NO has been demonstrated using PTIO as a scavenger molecule^24^. Following a simple chemical mechanism, NO is oxidized by PTIO to yield NO_2_ and 1-oxyl-2-phenyl-4, 4, 5, 5-tetramethylimidazoline (PTI) (Figure 1)^25^. The detection of PTI product can therefore serve to indicate the presence of NO in various samples. In addition, PTIO is also widely used for the quantitative detection of NO^26^. The selectivity for the conversion of PTIO to PTI in NO detection is high^27^, and the reaction of PTIO with NO forms solely PTI and NO_2_. Although NO_2_ can oxidize PTIO to PTIO^+^, quantitation of NO based on the yield of PTI is still valid, if an excess of PTIO is provided (this is the case in the current study, and the concentration of NO in breath is generally low)^28^. Usually, breath analysis by EESI-MS is done *in vivo*; i.e., volunteers donate their breath in front of a mass spectrometer in real time. However, the analysis of stationary patients with serious health problems, such as asthma, is difficult using this approach. To make our method more convenient for patients, the breath samples in this study were collected *in situ* by passing exhaled breath through a flow-control unit into collection bottles containing the PTIO solution (0.5 mg L^−1^). Following the sample collection, the resulting solution was directly sampled into EESI-MS for the targeted identification of PTI. By measuring the abundance of the characteristic PTI fragment (*m/z* 144) in the MS/MS detection mode, quantification of eNO content was achieved with a high analysis speed, sensitivity, and specificity. Instead of traditional ESI, EESI, a representative ambient ionization method^29^, was selected as the major detection tool because of its additional advantages of high tolerance to matrix effect and less contamination to the ion source, which helped to collect long-time reproducible and quantitative data.

## Results

### EESI-MS spectra of PTIO

Because PTIO is a radical molecule, it can produce various by-products during the ionization process. Therefore, EESI-MS analysis of pure PTIO solution provides an important reference/background check before the analysis of reaction products between PTIO and NO. In the EESI-MS spectrum recorded using a PTIO solution (0.5 mg L^−1^), abundant peaks were detected at *m/z* 203, 219, 234, 235, and 256 (Figure S-1a). The spectrum was dominated by the protonated PTIO ions [PTIO+H]^+^ (*m/z* 234), sodiated PTIO [PTIO+Na]^+^ (*m/z* 256), and even-electron [PTIO+2H]^+^ ions (*m/z* 235). EESI is an ambient ionization technique and NO has already been in the air, which might be the reason why PTI ions (*m/z* 218 and 219) were produced in Figure S-1a. However, these background signals were subtracted during the following concentration calculations.

In the EESI-MS/MS spectrum of [PTIO+H]^+^ (Figure S-1b), the major peaks at *m/z* 114 and *m/z* 84 were produced due to the cleavage of neutral C_7_H_6_NO and C_7_H_6_N_2_O_2_ from the precursor ions to generate [C_6_H_12_NO]^+^ ions and [C_6_H_12_]^+·^ ions, respectively. The precursor ions at *m/z* 234 also lost C_6_H_10_ to yield the fragment at *m/z* 152. In the MS^3^ analysis, the product ion at *m/z* 84 yielded major fragments at *m/z* 69 and *m/z* 56 (inset of Figure S-1b). Notably, the experimental parameters such as the ESI voltage, ESI solvent composition, ESI solvent injection rate, sample injection rate, ion-transport capillary temperature, and sheath gas (N_2_) pressure were experimentally optimized (detailed in Supplementary Information).

### EESI-MS analysis of PTI formed in the reaction between PTIO and NO gas standard

**Figure 2:**
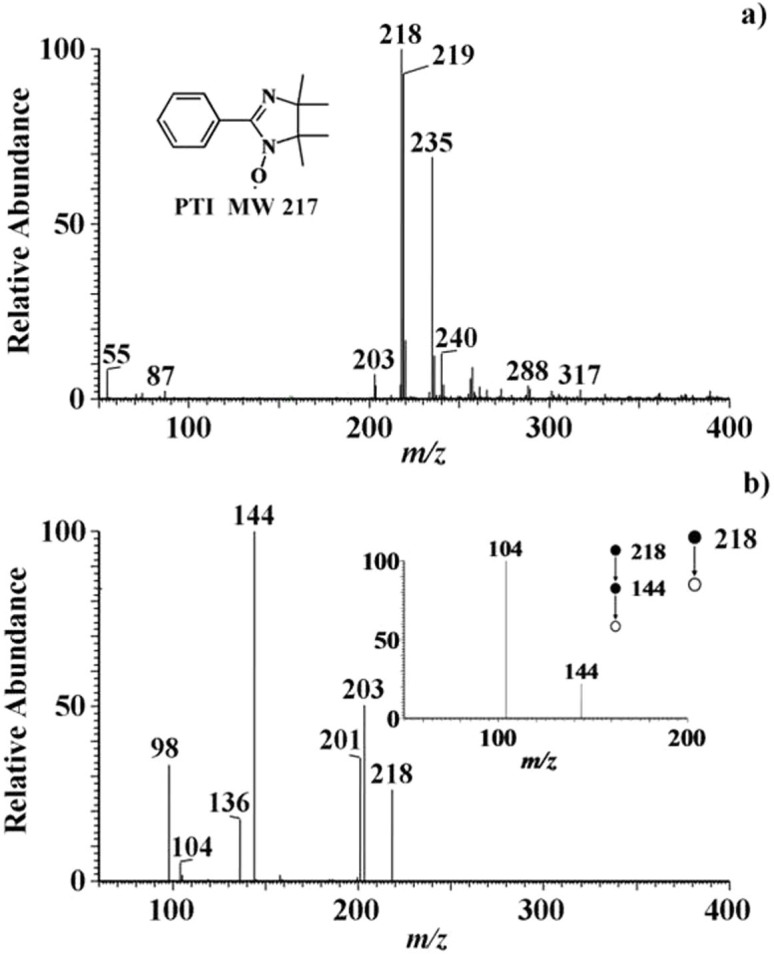
EESI-MS spectra of PTI. (a) Full scan EESI-MS mass spectrum of PTI (0.926 mg L^−1^); (b) MS^2^ spectrum of protonated PTI (*m/z* 218), and the inset shows the MS^3^ spectrum of *m/z* 218.

The EESI mass spectrum of PTI analyzed from the stock solution (0.926 mg L^−1^) was dominated by the protonated PTI ions [PTI+H]^+^ (*m/z* 218), while [PTI+2H]^+^ had a lower intensity (*m/z* 219) (Figure 2a). MS/MS experiment on the [PTI+H]^+^ ions generated major fragments at *m/z* 98, 104, 136, 144, 201, and 203 (Figure 2b). The precursor ions at *m/z* 218 also lost C_3_H_8_NO to yield the fragments at *m/z* 144 (potential chemical structures are shown in Figure S-2). The MS/MS spectrum of [PTI+2H]^+^ (*m/z* 219) (Figure S-3) and its possible fragmentation mechanism (Section 5) are shown in Supplementary Information.

### EESI-MS spectra of PTI after reaction with exhaled NO

The exhaled breath was passed through the PTIO solution to form PTI product as described in the Experimental section. To exclude the possibility of false-positive MS detection of PTI (Figure S-4a), the MS/MS pattern of *m/z* 218 ions from breath samples (Figure S-4b) were compared to that from the authentic PTI standard under the same experimental conditions (Figure 2b). The similarity between Figure S-4b and Figure 2b confirmed the successful detection of PTI.

### Calibration curves

To build calibration curves for PTIO and PTI in our experiments, each standard solution was analyzed 6 times independently under optimized experimental conditions (see Supplementary Information). For PTIO, the MS^2^ signal of *m/z* 84 after background subtraction was plotted as a function of PTIO concentration. The linear response range for PTIO was about 5 orders of magnitude (Figure S-5). The calibration equation was log_10_ I_1_ (intensity of *m/z* 84, cps) = 0.7181 log_10_ C_PTIO_ (concentration, ng L^−1^)-0.6131, with a linearity coefficient R^2^ = 0.995 using the logarithmic scale. The calibration curve for PTI was built as follows. Standard PTIO solutions were reacted with NO. The concentration of non-reacted PTIO remaining in each solution after the reaction was measured by EESI-MS. By subtracting the final concentration of PTIO from the original PTIO concentration in a standard sample, the amount of PTI product was determined. The PTI product in each reacted standard solution was then analyzed by EESI-MS. The [PTI+H]^+^ ion (*m/z* 218) was selected for MS/MS analysis, and the intensity of *m/z* 144 fragment was measured.

The calibration for PTI was converted to the calibration for eNO, based on their linear relationship: [eNO] (ppbv) = 565 [PTI] (mg L^−1^) (see Supplementary Information). Finally, a calibration curve was obtained for the concentration of eNO depending on the signal intensity of *m/z* 144. The linear equation in the log/log scale was: log_10_ [*m/z* 144, cps] = 1.0893 log_10_ [eNO, ppbv]+1.7133 (R^2^ = 0.995). The linear region for the calibration curve of eNO spanned 4 orders of magnitude (Figure S-6). The limit of detection (LOD, S/N = 3) was determined to be 0.02 ppbv. From six independent measurements the relative standard deviation (RSD) was estimated to be 11.6%.

### Dynamic change of NO level in the exhaled breath from volunteers before and after smoking

**Figure 3:**
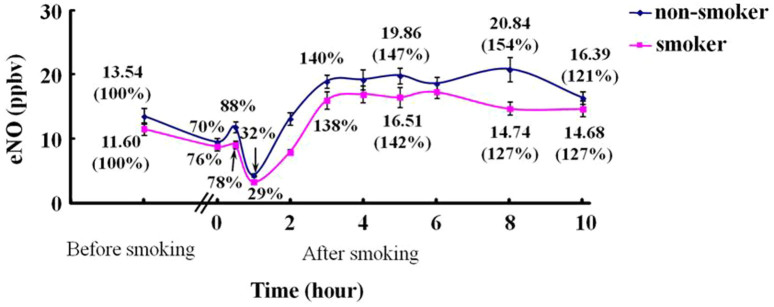
Dynamic change of NO levels in the exhaled breath of volunteers before and after smoking one cigarette.

Using the described method, we analyzed the dynamic change of NO observed in breath before and after smoking from 20 volunteers including 12 regular smokers and 8 non-smokers. The eNO concentration was plotted as a function of time after smoking a cigarette. Each data point in Figure 3 is the averaged percentage of the eNO concentrations to the first data point (before smoking) on the corresponding dynamic curve (concentration data/the first data on each dynamic curve × 100%). The recorded 10-h eNO profiles of all the subjects (Figure 3) showed that the mean eNO values of smokers and non-smokers before smoking (the first data points) were 11.6 ppbv and 13.5 ppbv, respectively. NO concentration decreased immediately after smoking down to a mean value of 8.8 ppbv (76%) for smokers and 9.5 ppbv (70%) for non-smokers, and the NO levels increased in about 30 min to 9.1 ppbv (78%) and 11.9 ppbv (88%), respectively. Then the NO concentration further decreased down to the lowest level at 3.3 (29%) ppbv (smokers) and 4.4 (32%) ppbv (non-smokers) in about 1 h. Finally, the NO level increased again in ca. 3 h. Furthermore, we examined the difference between smokers and non-smokers at each time point. It turned out that the most significant difference showed at t = 8 hrs with a Student's T-Test P value of 0.000072, and other time points had less difference (e.g., P = 0.16 at t = 3 hrs). In addition, we performed the spiking experiment using the breath samples from 5 subjects, which confirmed that our measurements were reliable. As shown in Table S-1, the average recovery rate was 102.6%, with a range of 99.3% to 106.3%.

## Discussion

The observation of abundant [PTIO+2H]^+^ ions (*m/z* 235, Figure S-1) in EESI-MS is probably due to the high reactivity of radical PTIO species. [PTIO+2H]^+^ can be formed by the direct attachment of one H^+^ and one H^·^ to PTIO^30^,^31^. The MS/MS spectrum of [PTIO+2H]^+^ (*m/z* 235) (Figure S-8) and its possible fragmentation mechanism (Section 4) are shown in Supplementary Information. The peaks at *m/z* 203 and *m/z* 219 correspond to the species formed via detachment of O_2_ and O from PTIO ions, respectively. These species are not observed in the MS/MS spectrum of PTIO ions and are therefore formed in solution rather than by fragmentation.

The fragments at *m/z* 201, 203, and 98 in the EESI-MS/MS spectrum of PTI product (Figure 2b) are due to the losses of OH, CH_3_, C_7_H_6_NO, respectively. The product ions at *m/z* 144 further fragment into *m/z* 104, probably by the loss of C_3_H_4_ (inset of Figure 2b). The relatively low intensity of PTI signal (compared to the intensity of PTI standard (0.926 mg L^−1^)) at *m/z* 218 produced by the reaction of PTIO with breath (Figure S-4a) reflects the usually low concentration of eNO in humans. We excluded the possibility of significant spectral interferences to the detection of PTI because the fragmentation pattern of *m/z* 218 in breath and reference samples were found to be nearly identical (Figure S-4b and 2b).

The recorded EESI-MS profiles indicate that right after smoking a cigarette the breath NO concentration is initially decreasing (Figure 3). The decrease of eNO within the first few minutes is consistent with the inhibition of nitric oxide synthase (NOS) activity by NO contained in cigarettes^32^,^33^. The follow-up increase of eNO observed for each individual at about 30 min agrees with the study by Chambers *et al*.^34^ The decrease of eNO signal can be explained by the reduced levels of NOS, the family of enzymes responsible for the NO production^35^,^36^. Reduced levels of stable end products of NO metabolism (e.g., nitrite and nitrate) have been reported in the saliva and serum of smokers 1 hr after smoking a single cigarette^37^. The oxidant molecules in cigarettes can also contribute to the decrease of NO in breath. Although the exact mechanism why smoking causes long-term fluctuation of eNO remains unclear, the capability of EESI-MS for quantitative detection of eNO makes it a potentially useful platform for more advanced studies of NO metabolism. Notably, the results of spiking experiments further validated that the present method is accurate for the eNO detection from human breath.

To conclude, a novel analytical method has been developed for the rapid and quantitative detection of NO in the exhaled breath with high chemical sensitivity and specificity based on the EESI-MS analysis of PTI produced during the reaction between NO and PTIO. In-situ sample collection makes our method applicable for stationary patients and convenient for large-population studies. The LOD for eNO detection is well below ppb level (~0.02 ppbv), and the relative standard deviation of measured eNO is 11.6%. Using the introduced method, characteristic time profile of eNO was investigated for smoking individuals. The exact mechanism by which smoking induces dynamic changes in the eNO level remains unknown, and the findings reported here offer an alternative analytical tool for advanced studies in this field.

## Methods

### Materials and Reagents

2-phenyl-4, 4, 5, 5-tetramethylimidazolineoxyl-1-oxyl-3-oxide (PTIO, >98.0%) was purchased from Tokyo Chemical Industry Co., Ltd. (Tokyo, Japan). The NO gas standard (10.2 × 10^3^ ppbv) was bought from Shenkai Gas Technology Co., Ltd. (Shanghai, China). Methanol (HPLC grade) was bought from ROE Scientific Inc. (Newark, DE, USA). Ultrapure water (18.2 MΩ·cm) was supplied by a Barnstead Nanopure ultrapure water purification system (Thermo Scientific, San Jose, CA, USA). The exhaled breath introduction line was made from the Teflon/polytetrafluoroethylene (PTFE) tube (ID 2 mm, OD 4 mm), which was purchased from Qiwei Industrial Material Co., Ltd. (Dongguan, China). The rotameter used to control the flow rate of exhaled breath was bought from Shenyang Zhengxing Flow Meter Co., Ltd. (Shenyang, China).

### Breath Samples

The methods were carried out in accordance with the approved guidelines, and all experimental protocols were approved by the Ethics Committee of the East China Institute of Technology and Nanchang University. All the subjects had been informed the content of this experiment before their breath tests, and informed consent was obtained from all subjects. Cigarettes used in the experiments were purchased from a local store. As noted by the manufacturer, a single dosage of cigarette produces 12 mg of tar, 13 mg of carbon monoxide, and 1.2 mg of nicotine. To examine the dynamic change of NO level in the exhaled breath, we collected breath samples from 20 volunteers, including 12 regular smokers and 8 non-smokers, before smoking and after (t = 0 hr, 0.5 hrs, 1 hr, 2 hrs, 3 hrs, 4 hrs, 5 hrs, 6 hrs, 8 hrs, 10 hrs). Volunteers for smoking experiments were chosen from healthy adults (age from 20–23). These 20 healthy adult volunteers maintained their normal daily lifestyle during the experiment periods, but were asked to refrain from smoking or exercise at least 6 hours before testing. The zero time point in the recorded PTI profiles corresponded to the moment when a volunteer just finished a cigarette. We also performed Student's T-Test to compare the difference between smokers and non-smokers at each time point. Spiking experiments were performed by bubbling diluted standard NO gas together with breath samples into the PTIO solution. The eNO concentration and the total NO concentration were detected respectively for calculating the recovery rates. Note that the experimental and safety issues have also been addressed according to the local legislation.

### Instrumentation and Working Conditions

All the experiments were carried out using a homemade EESI source coupled with a Thermo Finnigan hybrid LTQ-XL mass spectrometer (San Jose, CA, USA). The EESI source design and principle were detailed elsewhere^21^,^22^,^38^. The LTQ mass analyzer was operated in the positive-ion detection mode, and mass spectra were recorded in the range *m/z* 50–400. The ESI high voltage was set at +3.0 kV. The temperature of the ion entrance capillary was 300°C. Nitrogen gas (purity ≥ 99.999%) was supplied at a pressure of 1.4 MPa. ESI solvent (methanol) and the sample solution were supplied at a flow rate of 5 μL min^−1^ and 6 μL min^−1^, respectively. The angle between the two spray channels in EESI was 60°. The distance between the sprayer tips was 1.5 mm. The angle between each sprayer and MS inlet capillary was 150°. The distance from the tip of each sprayer to the MS inlet was 5 mm.

In the collision induced dissociation (CID) experiments of PTIO and PTI, the protonated ions at *m/z* 234 and *m/z* 218 were selected as the precursor ions, respectively. The isolation width was 1.2 Da and the collision activation time was 30 ms, with 19–32% (arbitrary units defined by the LTQ instrument) collision energy. The maximum ion injection time was 100 ms and the Automatic Gain Control (AGC) was enabled to regulate the number of ions injected into the ion trap. Other parameters were automatically optimized by the LTQ-MS system.

### Preparation of standard PTIO and PTI solutions

The stock PTIO solution was prepared by dissolving 10 mg of PTIO into 100 mL of methanol, and then it was stored in the 100-mL brown volumetric flask in the dark. Standard working solutions (0.0001–5 mg L^−1^) were prepared by diluting the PTIO stock solution with methanol.

The stock solution of PTI (0.926 mg L^−1^) was prepared by reacting 10 mL of PTIO (1.0 mg L^−1^) with NO gas (10.2 × 10^3^ ppbv) for about 30 minutes. The stock solution of PTI was diluted serially with methanol to give final dilutions in the range of 1/2.5 to 1/10000 mg L^−1^.

### Collection of exhaled NO samples

10 mL of standard PTIO solution (0.5 mg L^−1^) was added into an empty and dry brown glass reagent bottle. Exhaled NO samples were prepared by bubbling the exhaled breath directly through the PTIO solution (0.5 mg L^−1^) *via* a PTFE tube at a stable exhalation flow rate which was controlled by a rotameter, and the controlled flow rate was 0.8 L min^−1^. Each sample was collected for 150 s (10 successive 15-s samplings). The collected samples were directly analyzed by EESI-MS without further treatment. The experiments were performed at 25°C.

## Author Contributions

S.P., Y.T. and H.G. designed the project, wrote, and revised the manuscript. M.L., X.F., H.W., J.S. and W.Z. gave useful comments and intellectual support, J.Z. and L.Z. performed volunteers' sample detection and data processing, P.L. and H.C. provided important assistance in revising the manuscript. All authors reviewed manuscript.

## Supplementary Material

Supplementary Information Detection of eNO by EESI-MS Supplementary Information

## Figures and Tables

**Figure 1:**
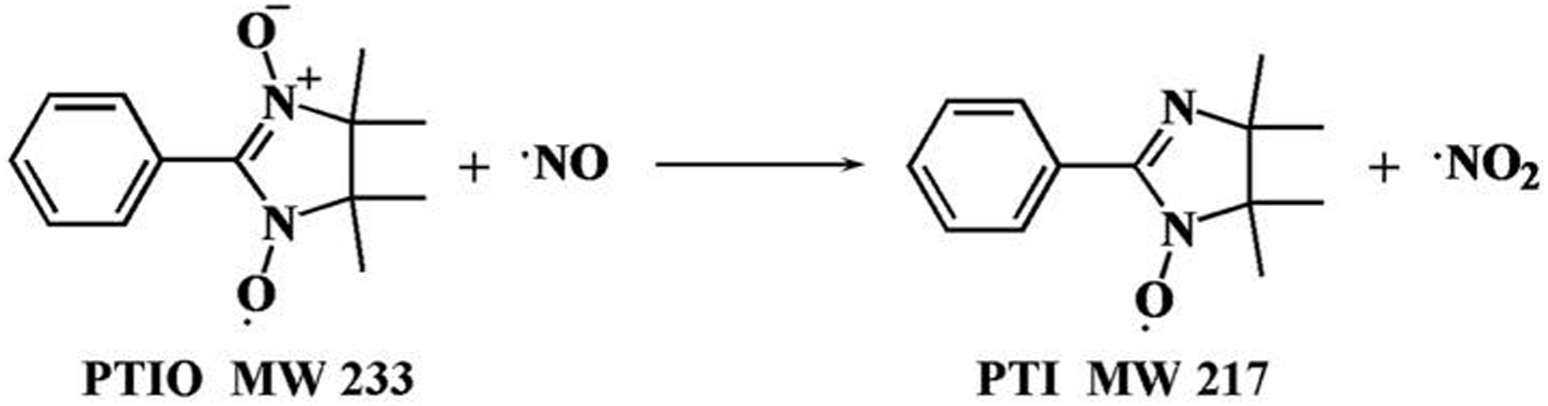
The reaction of PTIO with NO.
