# Supplementary material for: Quantitative detection of nitric oxide in exhaled human breath by extractive electrospray ionization mass spectrometry
Source: Sci Rep. 2015 Mar 4;5:8725. doi: 10.1038/srep08725 (PMC4348632; doi:10.1038/srep08725)
Supplement: Supplementary Information — Detection of eNO by EESI-MS Supplementary Information [file srep08725-s1.pdf]

Supplementary Information

Quantitative detection of nitric oxide in exhaled human breath by extractive electrospray ionization mass spectrometry

Susu Pan[a†], Yong Tian[b†], Ming Li[c], Jiuyan Zhao[d], Lanlan Zhu[d], Wei Zhang[d], Haiwei Gu[a*], Haidong Wang[a], Jianbo Shi[b], Xiang Fang[c], Penghui Li[a], Huanwen Chen[a]

[a] *Jiangxi Key Laboratory for Mass Spectrometry and Instrumentation, East China Institute of Technology, Nanchang, Jiangxi Province 330013, P. R. China*

[b] *State Key Laboratory of Environmental Chemistry and Ecotoxicology, Research Center for Eco-Environmental Sciences, Chinese Academy of Sciences, Beijing 100085, P. R. China*

[c] *National Institute of Metrology, Beijing 100013, P. R. China*

[d] *Department of Respiratory Medicine, The First Affiliated Hospital of Nanchang University, Nanchang, Jiangxi Province 330006, P. R. China*

[†]: These authors contributed equally to this work.

*: Corresponding author: Dr. Haiwei Gu

E-mail: guhaiwei2004@gmail.com; Tel/fax: 86-0791-83896370

Experimental Details

1. Calculation procedure for the mass concentration of the stock solution of PTI (C_{PTI})

After reaction with a NO gas standard, the concentration of surplus PTIO can be calculated according to the calibration curve of PTIO. One mole of NO is oxidized by PTIO to yield one mole of NO_2 and one mole of PTI. Hence,

$$n_{PTI} = n_{PTIO} \tag{1}$$

where n_{PTI} is the number of moles (mol) of PTI produced; n_{PTIO} is the number of moles of PTIO consumed. Entering the mass concentration of stock solution of PTIO (1.0 mg L^{-1}) to eqn. (1), the n_{PTIO} can be calculated as below:

$$n_{PTIO} = \frac{\left(0.001 g L^{-1} - C_{PTIO}\right) \times V_{PTIO}}{m_w} \tag{2}$$

Entering eqn. (2) into eqn. (1), the number of moles of PTI produced can be calculated using the equation below:

$$\begin{aligned} n_{PTI} &= n_{PTIO} \\ &= \frac{\left(0.001 g L^{-1} - C_{PTIO}\right) \times V_{PTIO}}{m_w} \end{aligned} \tag{3}$$

where C_{PTIO} is the mass concentration ($g L^{-1}$) of remnant PTIO after reaction with a NO gas standard, C_{PTIO} shall be calculated according to the calibration curve of PTIO, V_{PTIO} is the volume of PTIO solution, and m_w is the molar mass of PTIO.

Given

$$V_{PTIO} = 0.01L\,;\quad m_w = 233.3\,g\,mol^{-1}$$

and entering these into eqn. (3), thus

$$n_{PTI} \approx \left[4.29\times10^{-5}\left(0.001\,g\,L^{-1} - C_{PTIO}\right)\right]mol \tag{4}$$

So the mass concentration ($g\,L^{-1}$) of the stock solution of PTI after reaction with a NO gas standard can be calculated as below:

$$C_{PTI} = \frac{n_{PTI}\times m_n}{V_{PTI}} \tag{5}$$

where V_{PTI} is the volume of the mixture solution after reaction. Actually, m_n is the molar mass of PTI: $m_n = 217.3\,g\,mol^{-1}$, and $V_{PTI} = V_{PTIO} = 0.01L$. Entering m_n, V_{PTI}, and eqn. (4) into eqn. (5), then

$$\begin{aligned} C_{PTI} &= \frac{217.3\,g\,mol^{-1}\times n_{PTI}}{0.01L} \\ &= \frac{217.3\,g\,mol^{-1}\times\left[4.29\times10^{-5}\left(0.001\,g\,L^{-1} - C_{PTIO}\right)\right]mol}{0.01L} \\ &= 0.932\times\left(0.001\,g\,L^{-1} - C_{PTIO}\right) \end{aligned} \tag{6}$$

So the mass concentration of the stock solution of PTI (C_{PTI}) can be calculated according to eqn. (6).

2. Calculation procedure of eNO

For exhaled NO samples, the number of moles of NO also equals to n_{PTI} according

to the proportion relation of the reaction:

$$n_{NO} = n_{PTI} \tag{7}$$

From eqn. (5), n_{PTI} can be calculated as below:

$$n_{PTI} = \frac{C_{PTI}{}' \times V_{PTI}}{m_n} \tag{8}$$

where $C_{PTI}{}'$ is the mass concentration ($g\,L^{-1}$) of PTI produced after reaction with NO in breath. Entering eqn. (8) into (7), then

$$n_{NO} = \frac{C_{PTI}{}' \times V_{PTI}}{m_n} \tag{9}$$

Given

$$V_{PTI} = 0.01L;\ m_n = 217.3\,g\,mol^{-1}$$

and entering these into eqn. (9), thus

$$\begin{aligned} n_{NO} &= \frac{C_{PTI}{}' \times 0.01L}{217.3\,g\,mol^{-1}} \\ &= \left(4.6\times10^{-5} C_{PTI}{}'\right) mol \end{aligned} \tag{10}$$

$$V_{NO} = n_{NO} \times V_m \tag{11}$$

According to the Clapeyron equation, at a temperature of 25 ℃ and pressure of 1.01×10^5 Pa, the volume of 1 molar NO can be approximately calculated as below:

$$
\begin{aligned}
V_m &= \frac{nRT}{P} \\
&= \frac{1mol \times 8.314\,J\,K^{-1} mol^{-1} \times 298K}{1.01 \times 10^5\,Pa} \\
&\approx 24.5 \times 10^{-3}\,m^3 \\
&= 24.5\,L
\end{aligned}
$$

Entering $V_m = 24.5\,L\,mol^{-1}$ and eqn. (10) into eqn. (11), then

$$
\begin{aligned}
V_{NO} &= n_{NO} \times V_m \\
&= 24.5\,L\,mol^{-1} \times n_{NO} \\
&= 24.5\,L\,mol^{-1} \times \left(4.6 \times 10^{-5} \times C_{PTI}{}'\right) mol \\
&= \left(1.13 \times 10^{-3} C_{PTI}{}'\right) L
\end{aligned}
\tag{12}
$$

Because

$$
eNO = \frac{V_{NO}}{V} \tag{13}
$$

where V is the total volume (15 s for each exhalation, 10 times) of exhaled breath bubbling through the PTIO solution, and the controlled flow rate is 0.8 L min^{-1}, then

$$
\begin{aligned}
V &= \frac{15}{60} \min \times 10 \times 0.8\,L \min^{-1} \\
&= 2\,L
\end{aligned}
$$

Entering $V = 2\,L$ and eqn. (12) into eqn. (13), then

$$
\begin{aligned}
eNO &= \frac{V_{NO}}{V} \\
&= \frac{\left(1.13 \times 10^{-3} C_{PTI}{}'\right) L}{2\,L} \\
&= \left(5.65 \times 10^{5}\, C_{PTI}{}'\right) \text{ppbv} \qquad \left(1\text{ppbv} = 1.0 \times 10^{-9}\right)
\end{aligned}
\tag{14}
$$

Consequently, eNO was calculated using eqn. (14). The eqn. (14) also shows that eNO is proportional to C_{PTI}'.

3. Optimization of experimental parameters

To achieve better sensitivity for NO detection, the signal intensities of $[PTI+H]^+$ (*m/z* 218) and its characteristic fragment (*m/z* 144) were optimized by adjusting the ESI voltage, ESI solvent composition, ESI solvent injection rate, sample injection rate, ion-transport capillary temperature, and sheath gas (N_2) pressure. The data obtained using either PTIO or PTI showed the same optimal conditions; thus the discussion in this section is focused on the optimization of PTIO signal, and the similar data obtained using PTI are not shown.

ESI voltage. The effect of electrospray voltage on the signal intensity of characteristic fragment (*m/z* 84) of PTIO is shown in Figure S-7a. The data indicated that the optimal ESI voltage to detect PTIO was in the range 1.0-3.0 kV. The highest signal intensity for the *m/z* 84 fragment was obtained at 3 kV. When a voltage higher than 3.5 kV was used, a corona discharge between the two channels of the EESI source was occasionally observed. This resulted in lower efficiency of ionization and the decreased signal intensity of PTIO signal.

ESI solvent composition. For optimizing the primary ESI solvent composition, different proportions of methanol/water were tested. The highest intensity was obtained when 100% methanol was applied (Figure S-7b). This can be due to the

higher desolvation efficiency of methanol than water.

ESI solvent injection rate. The signal intensity level of characteristic fragment *m/z* 84 was affected by the ESI solvent injection rate, and the result is shown in Figure S-7c. For low flow rates (1–5 L min^{-1}), the signal intensity increased with the increase of the ESI solvent injection rate. However, the signal intensity started to decrease when the injection rate exceeded 5 L min^{-1}, probably because the nebulization efficiency was affected by the higher flow rates.

Sample injection rate. The signal intensity of characteristic fragment (*m/z* 84) of PTIO was also affected by the sample injection rate (neutral channel in EESI), and the result is shown in Figure S-7d. When the sample injection rate was raised from 1 L min^{-1} to 6 L min^{-1}, the signal intensity was increased. However, the signal was dropped when the sample injection rate exceeded 6 L min^{-1}. Because higher injection rates can cause chemical contamination to the inlet of mass spectrometer, the sample injection rate in this work was finally adjusted to 6 L min^{-1}.

Ion-transport capillary temperature. The desolvation process of charged droplets can be facilitated by elevated temperature of the ion-transport capillary, resulting in a better efficiency of producing gaseous species. When the temperature of ion-transport capillary of the LTQ instrument was increased from 100 to 300 ℃, the signal intensity of *m/z* 84 was rapidly increased, possibly due to the better desolvation effects (Figure S-7e). However, the signal intensity decreased slightly when the ion-transport capillary temperature was higher than 300 ℃, which can be due to the thermal

dissociation of PTIO/PTI ions inside the ion-transport capillary.

Sheath gas pressure. The pressure of sheath gas affected the signal intensity of characteristic fragment *m/z* 84. The result is shown in Figure S-7f. Below the optimal pressure of 1.4 MPa, sample nebulization was poor, leading to a lower signal intensity of *m/z* 84. The signal decrease at higher pressures can be due to the decreased efficiency of online liquid-liquid extraction/ionization in the EESI plume, since faster droplets have lower residence time in the extraction area.

4. MS/MS analysis of $[PTIO+2H]^+$ (*m/z* 235)

Upon CID, the ions $[PTIO+2H]^+$ (*m/z* 235) generated fragments at *m/z* 201, 153, 105, 98, 84 and 83 (Figure S-8). The largest fragment (*m/z* 201) was produced by the loss of H_2O_2 from the precursor ions. The MS^3 spectrum of the ions at *m/z* 201 (inset of Figure S-8) showed that the precursor ions (*m/z* 201) decomposed into the fragment ions at *m/z* 144 and *m/z* 98 through the loss of C_3H_7N and C_6H_5CN, respectively. The low abundant ions at *m/z* 104 which were observed in the MS^3 spectrum of precursor ions (*m/z* 201) were attributed to the fragmentation of the ions at *m/z* 144. The other fragments (*m/z* 153, 105, 84, and *m/z* 83) observed in MS/MS spectrum of the ions $[PTIO+2H]^+$ were produced by the loss of C_6H_{10}, $C_6H_{14}N_2O$, $C_7H_7N_2O_2$, and $C_7H_8N_2O_2$, respectively. The MS^2 spectrum of *m/z* 235 showed product ion signals at *m/z* 105 and *m/z* 84, which corresponded to benzoyl ions and $C_6H_{12}^{+\cdot}$ ions, respectively.

5. MS/MS analysis of [PTI+2H]$^+$ (*m/z* 219)

In the MS^2 spectrum of [PTI+2H]$^+$, precursor ions *m/z* 219 generated product ions of *m/z* 145, 137, 119, 98, and 83, as shown in Figure S-3. The ions of *m/z* 137 were obtained by ring-opening of precursor ions *m/z* 219 through loss of C_6H_{10}. Signals at *m/z* 120, 119, and 104 in MS^3 spectrum were observed by loss of $OH^{\cdot}$, H_2O, and NH_2OH, respectively. PTIO is a five membered nitrogen containing heterocyclic compound, and the ions *m/z* 98 observed in MS^2 spectrum were probably generated by loss of $C_6H_5CH_2NO$ from *m/z* 219 through cleavage of C=N bond followed by rearrangement. Ring-opening and reduction of C=N bond in imidazoles could happen with assistance of special catalyst. Therefore the abundance of *m/z* 98 was relatively low as the efficiency of ring-opening is very low. The product ions of *m/z* 145 and *m/z* 83 in MS^2 spectrum of ions at *m/z* 219 were generated by loss of C_3H_8NO and $C_7H_8N_2O$, respectively.

Table S-1. The results of spiking experiment using the breath samples from from 5 subjects.

	Base value (ppbv)	Spiked (ppbv)	Total (ppbv)	Recovery (%)
Subject 1	11.5 ±0.1	9.5	21.6 ±1.0	106.3
Subject 2	11.1 ±0.3	9.5	21.0 ±0.5	104.2
Subject 3	9.3 ±0.2	9.5	18.9 ±0.8	101.0
Subject 4	11.7 ±0.3	42.1	54.7 ±0.4	102.1
Subject 5	21.1 ±1.2	42.1	62.9 ±0.3	99.3

Figures

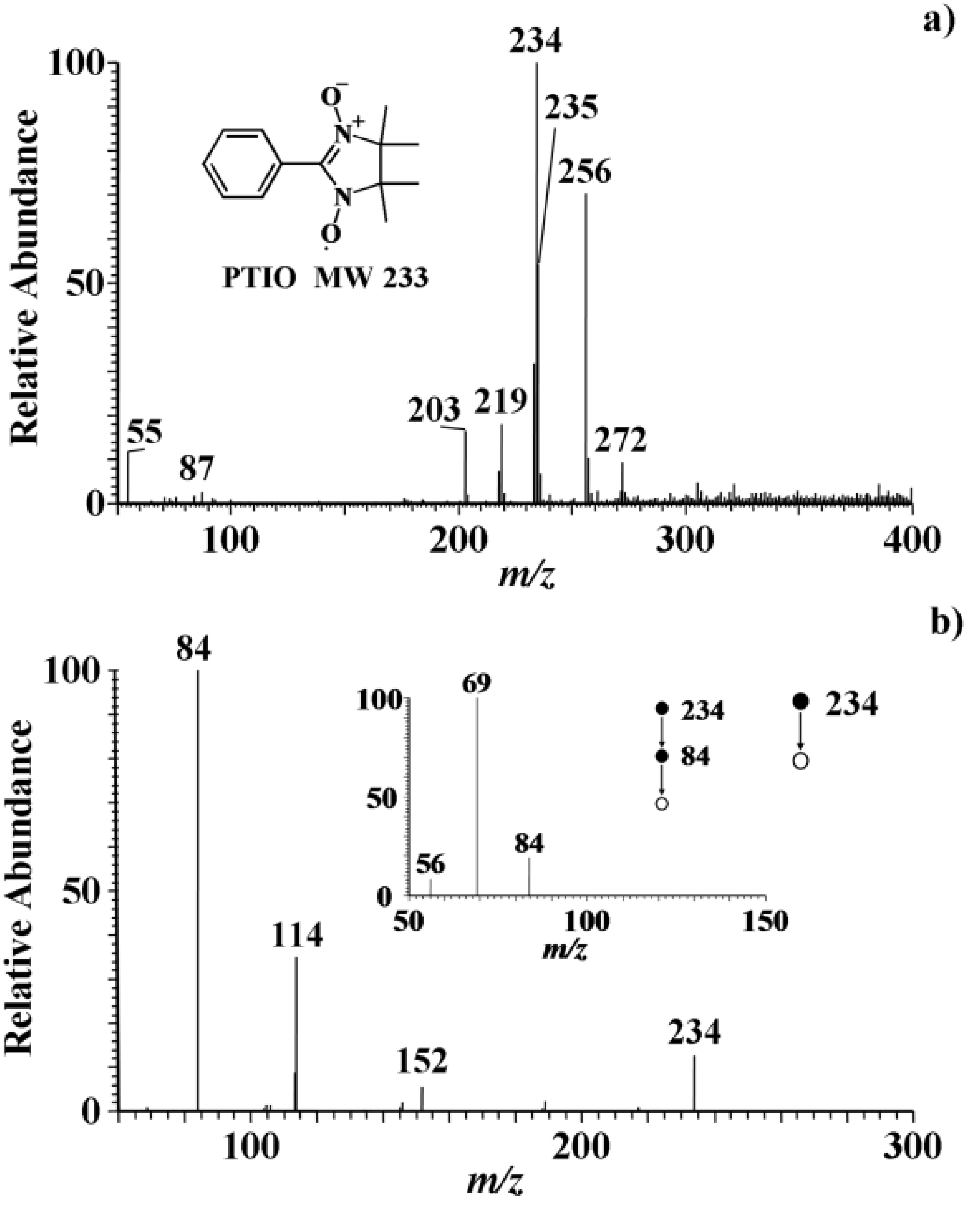

Figure S-1. EESI-MS spectra of PTIO. a) Full scan EESI-MS mass spectrum of PTIO (0.5 mg L^{-1}); b) MS^2 spectrum of protonated PTIO (*m/z* 234), and the inset shows the MS^3 spectrum of *m/z* 234.

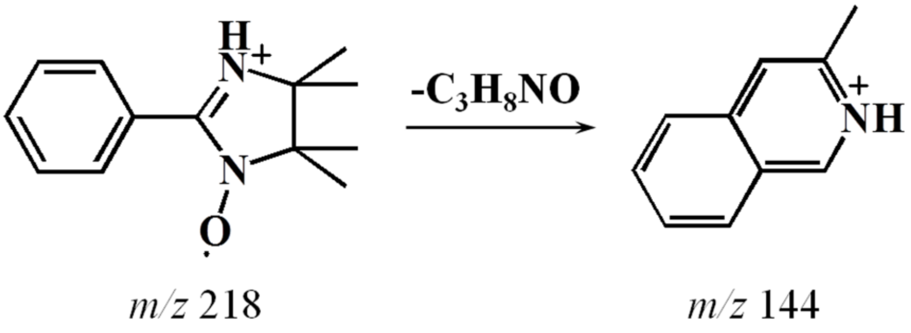

Figure S-2. Proposed structure for the product ions from protonated PTI in positive ion detection mode ($[PTI+H]^+$, *m/z* 218).

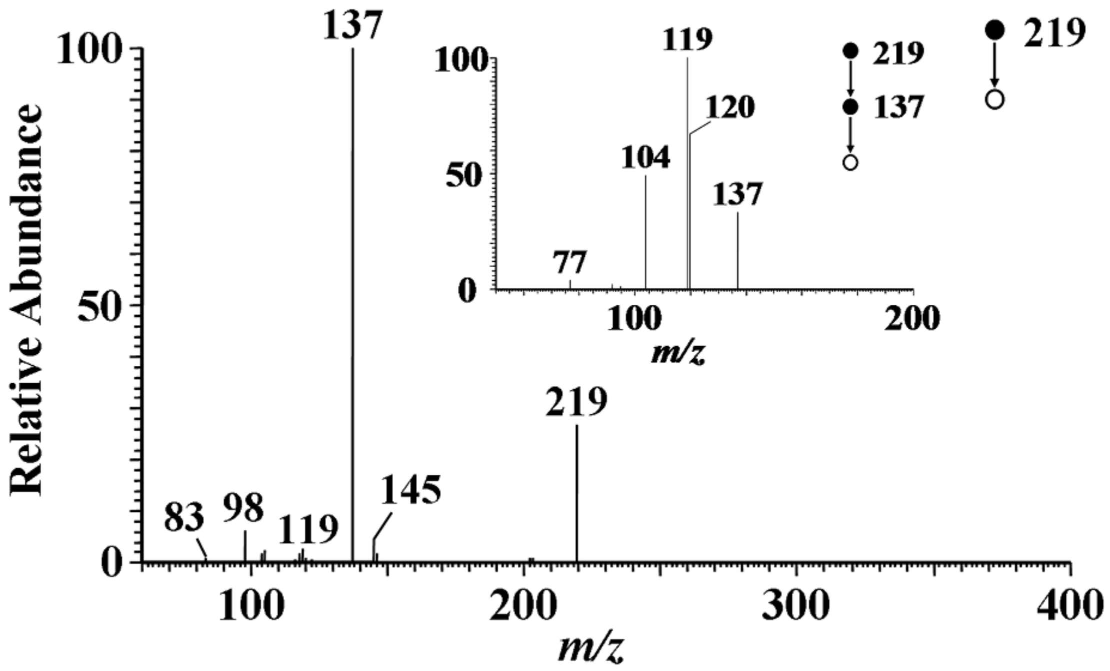

Figure S-3. MS^2 spectrum of $[PTI+2H]^+$ (*m/z* 219). The inset shows the MS^3 spectrum of ions of *m/z* 219. See MS/MS analysis of $[PTI+2H]^+$ (*m/z* 219) in Section 5.

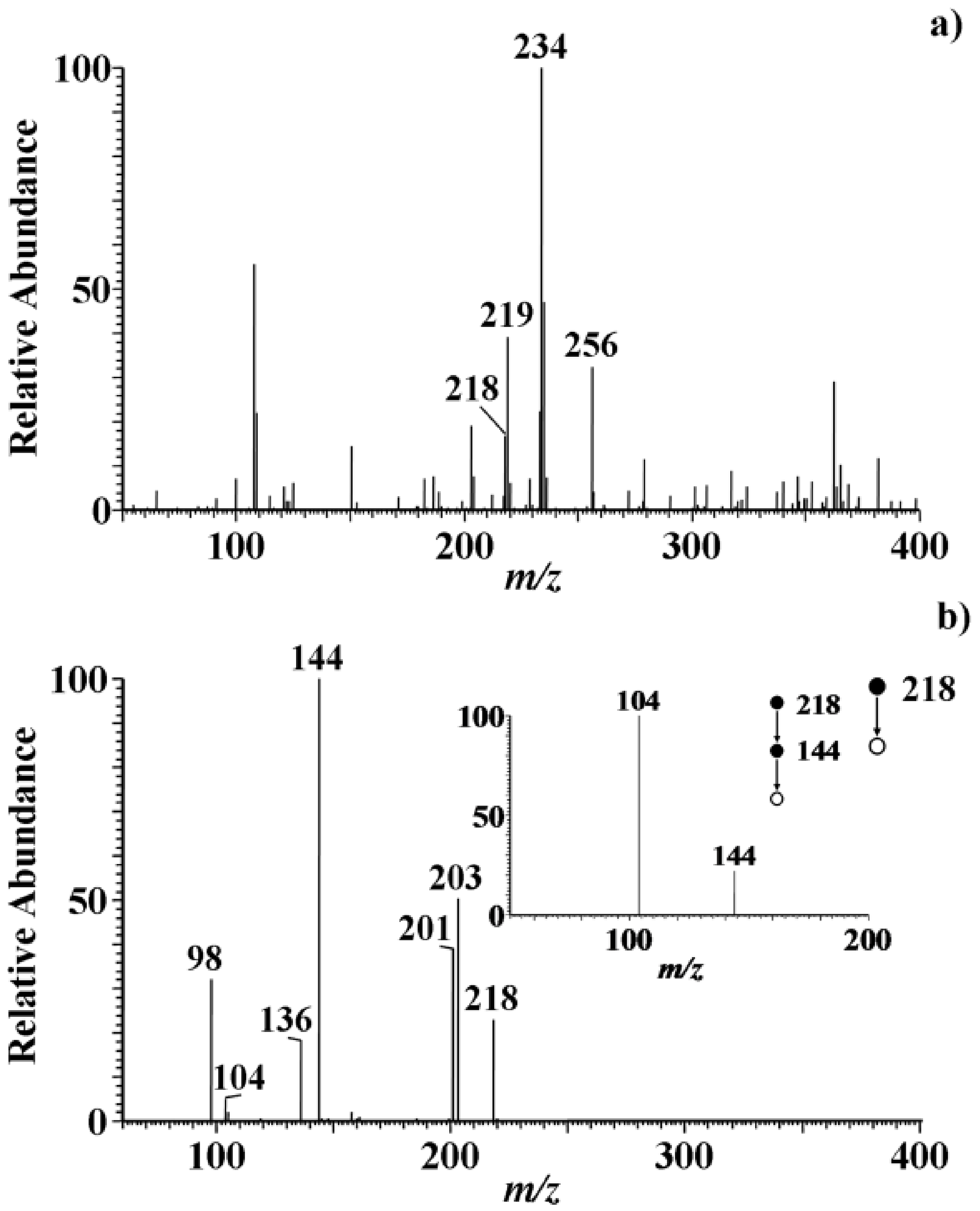

Figure S-4. Detection of PTI, the resultant of PTIO, and exhaled NO, by EESI-MS. a) PTI signal (*m/z* 218); b) MS^2 spectrum of *m/z* 218, and the inset shows the MS^3 spectrum of *m/z* 218.

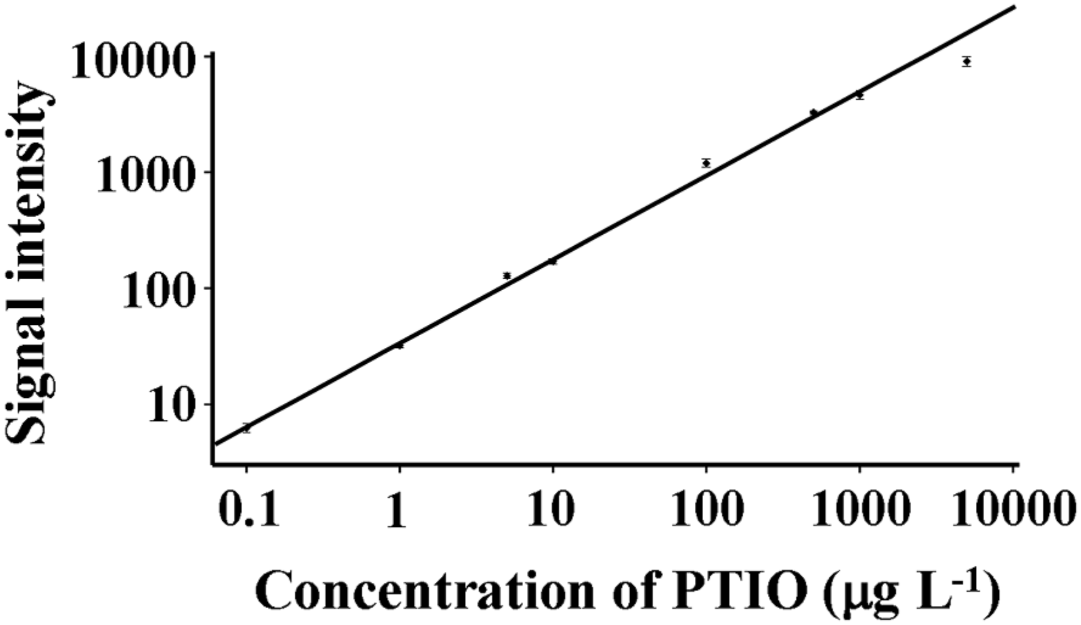

Figure S-5. Calibration curve of PTIO obtained using EESI-LTQ-MS.

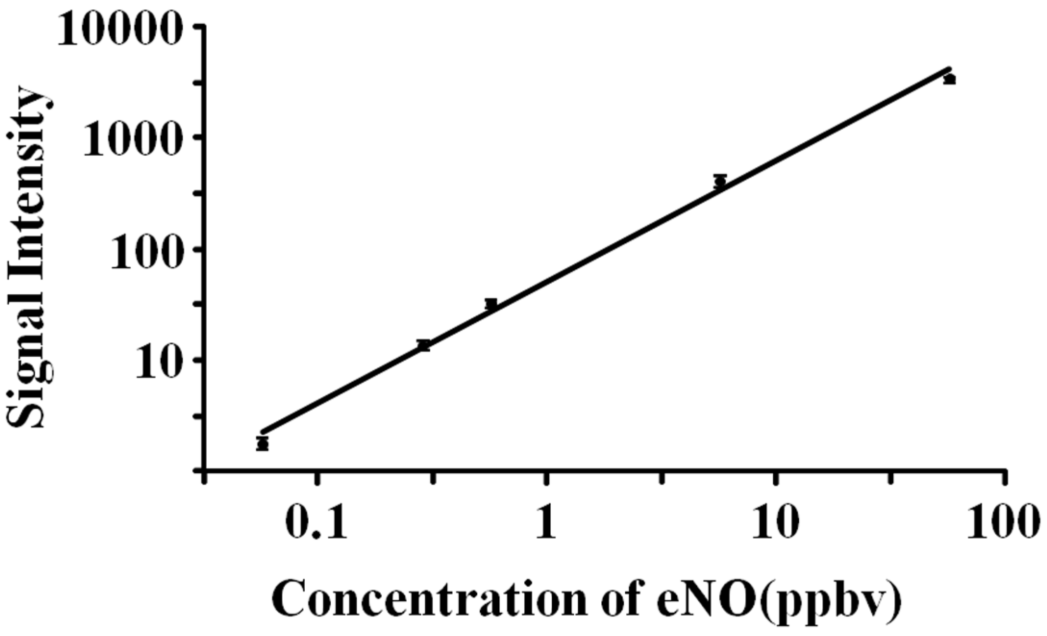

Figure S-6. Calibration curve for the detection of NO concentration obtained using EESI-LTQ-MS analysis of PTI product for the reaction between NO and PTIO.

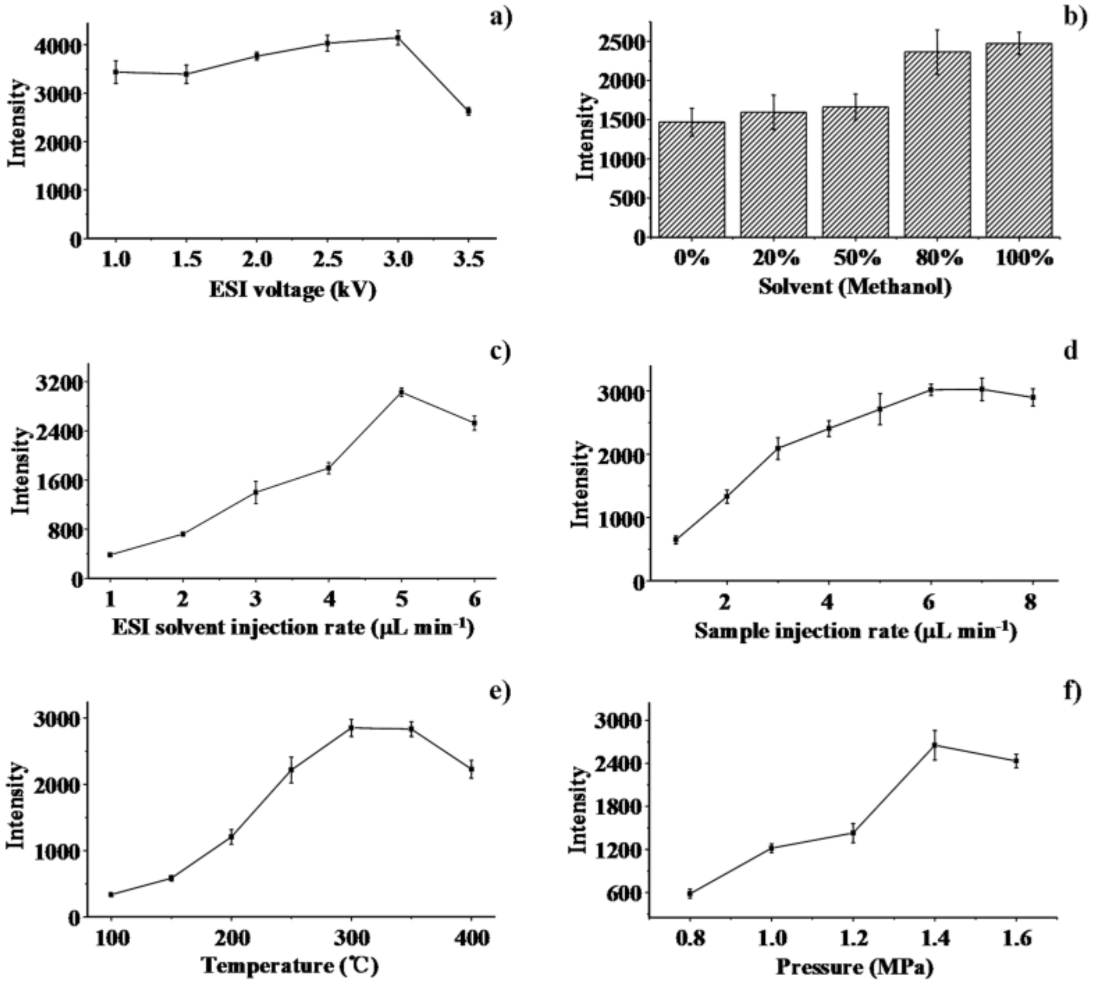

Figure S-7. Optimization of experimental parameters for EESI. a) ESI voltage, b) composition of ESI solvent, c) ESI solvent injection rate, d) sample injection rate, e) ion-transport capillary temperature, and f) nebulizing gas (N_2) pressure.

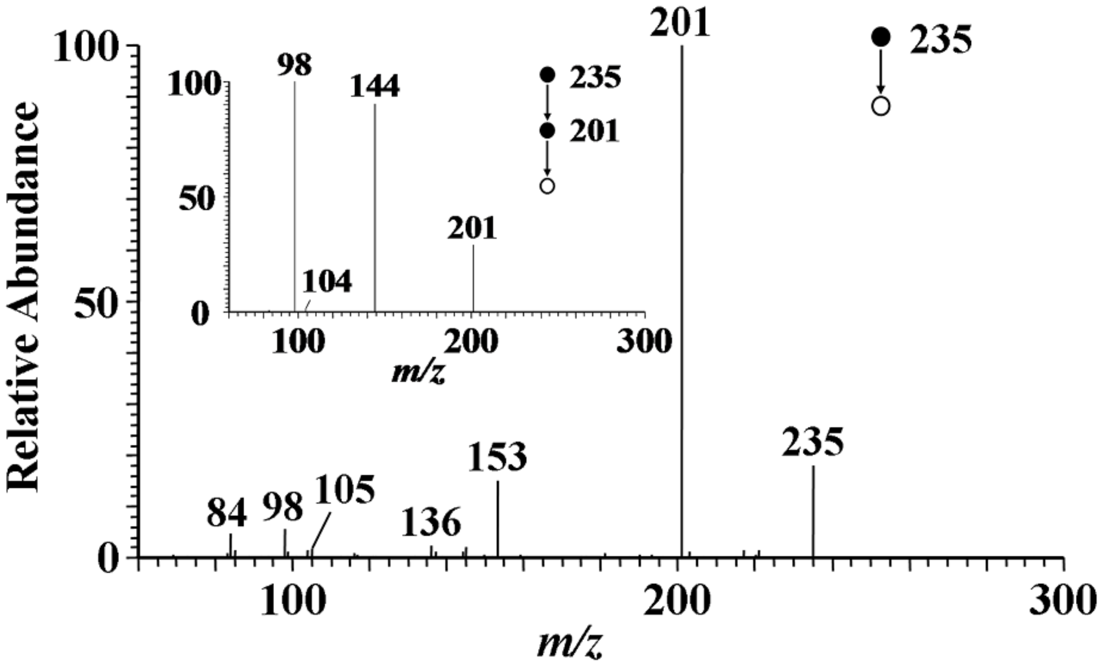

Figure S-8. MS^2 spectrum of $[PTIO+2H]^+$ (*m/z* 235). The inset shows the MS^3 spectrum of ions of *m/z* 235. See MS/MS analysis of $[PTIO+2H]^+$ (*m/z* 235) in Section 4.
